# Implementation of Organ Preservation for Locally Advanced Rectal Cancer in Canada: A National Survey of Clinical Practice

**DOI:** 10.3390/curroncol32060341

**Published:** 2025-06-10

**Authors:** Megan Delisle, Victoria Ivankovic, Doris Goubran, Eliane Yvonne Paglicauan, Mariam Alsobaei, Nicole Alcasid, Mary Farnand, Kristopher Dennis

**Affiliations:** 1 Department of Surgery, University of Manitoba, Winnipeg, MB R3E 0W3, Canada; alsobaem@myumanitoba.ca; 2 Department of Surgery, University of Ottawa, Ottawa, ON K1N 6N5, Canada; vivankovic@toh.ca; 3 Max Rady College of Medicine, University of Manitoba, Winnipeg, MB R3E 0W2, Canada; goubrand@myumanitoba.ca; 4 Faculty of Science, University of Manitoba, Winnipeg, MB R3T 2N2, Canada; paglicay@myumanitoba.ca (E.Y.P.); alcasidn@myumanitoba.ca (N.A.); 5 Ottawa Regional Cancer Program, The Ottawa Hospital, Ottawa, ON K1H 8L6, Canada; mafarnand@toh.ca; 6 Division of Radiation Oncology, University of Ottawa, Ottawa, ON K1H 8L6, Canada; krdennis@toh.ca

**Keywords:** rectal cancer, organ preservation, watch and wait, neoadjuvant therapy, oncology

## Abstract

Purpose: Organ preservation (OP) is an increasingly recognized treatment for locally advanced rectal cancer. However, variability in patient selection, treatment regimens, and surveillance can hinder widespread adoption. We conducted a national, cross-sectional survey evaluating how OP is implemented across Canada. Methods: Between June and July 2023, surgeons, radiation oncologists, and medical oncologists with expertise in OP from all 44 Canadian radiation centers completed an electronic survey about the implementation of OP at their centers. Primary OP was defined as administering neoadjuvant therapy with the explicit goal of avoiding surgery. Secondary OP was defined as deferring planned surgery only when there was an unexpected yet sufficient clinical response. Results: Responses from 40 radiation centers (response rate 90.9%) identified that 20 (50.0%) offered primary and secondary OP, 11 (27.8%) offered only secondary, and 8 (20.0%) offered neither. The most common treatment in primary OP was chemoradiation with consolidation chemotherapy (17/20, 89.5%). When assessing the response in primary OP, endoscopic biopsies were more commonly performed with a near-complete response (10/20, 50.0%) than a complete response (4/20, 20.0%). Watch-and-wait surveillance was more frequently offered for a complete response (17/31, 54.8%) than a near-complete response (4/31, 12.9%). Challenges included limited MRI (21/40, 52.5%), clinic time (18/40, 45.0%), timely surgery if required (16/40, 40.0%), and limited familiarity with the protocols and evidence (15/40, 37.5%). Conclusion: OP is recognized nationwide but is inconsistently implemented. Challenges raise concerns about the current feasibility of OP in Canada and highlight the need for less resource-intensive, complex OP protocols.

## 1. Introduction

Standard care for locally advanced rectal cancer (stage II/III, T1-2N+, T3/4Nany) typically involves neoadjuvant chemoradiation followed by total mesorectal excision (TME). This approach has lowered local recurrence rates and increased sphincter preservation [1]. Yet, postoperative morbidity and long-term functional problems (e.g., urinary, sexual, and anorectal dysfunction) are common, significantly affecting patients’ quality of life [2]. Recognizing that some patients achieve a complete pathological response after neoadjuvant treatment has prompted interest in deferring surgery for those with no residual tumor—a strategy known as organ preservation or “watch and wait” [3].

Organ preservation is delivered with the specific goal of achieving a clinical complete response (cCR) or near-complete response (nCR) after neoadjuvant therapy, thereby avoiding TME. Patients who achieve a cCR or nCR after neoadjuvant treatment may choose close surveillance (watch and wait) rather than immediate surgery. Growing evidence suggests organ preservation can be safe and effective in selected patients. Indeed, multiple guidelines endorse organ preservation for locally advanced rectal cancer [4,5,6,7,8,9]. However, significant heterogeneity persists in patient selection, neoadjuvant regimens, response assessment, and surveillance strategies, hindering broad adoption in everyday practice [5,10]. Understanding the gaps and challenges in the implementation of organ preservation is needed to inform future studies and quality improvement efforts.

In Canada, the implementation of organ preservation for locally advanced rectal cancer is largely unknown. Our national cross-sectional survey aimed to describe the current implementation of organ preservation in the 44 Canadian cancer centers offering definitive treatment for rectal cancer. We sought to assess the extent of organ preservation, identify challenges in its use, and highlight areas for improvement to inform equitable access for Canadian patients.

## 2. Methods

This study follows the Consensus-Based Checklist for Reporting of Survey Studies (CROSS) guidelines [11]. The protocol was approved by the Institutional Review Board of The Ottawa Hospital (OHSN-REB Number 20230275-01H).

### 2.1. Study Design and Setting

We conducted our study between April and October 2023. In Canada, rectal cancer care is coordinated exclusively through publicly funded regional centers that offer access to radiation therapy, systemic therapy, and surgery. There are 44 such centers. Hence, these 44 sites represent the entirety of Canadian centers delivering definitive (i.e., curative intent) treatment for rectal cancer.

### 2.2. Sample Characteristics

We used non-probabilistic convenience sampling to identify “key informants” at each of the 44 centers. It is unlikely that patients would receive rectal cancer care at different centers that were not sampled. 

Key informants were defined as clinicians (surgeons, medical oncologists, or radiation oncologists) with expertise in locally advanced rectal cancer management and organ preservation at their centers. This approach follows standard qualitative research methods for identifying individuals with deep knowledge of a specific practice environment [12]. We used existing professional networks and records from the Canadian Association of Radiation Oncology (CARO) to find regional experts. CARO records the department heads of all certified radiation centers in Canada, including academic and non-academic centers. If no expert was readily identified, we employed snowball sampling. Initial contacts were asked to suggest colleagues who might meet the study criteria, namely, clinical expertise in managing locally advanced rectal cancer and organ preservation strategies. On average, we contacted two or three clinicians per site to find a single key informant for each center.

### 2.3. Data Collection Methods

We developed an electronic survey using previously published tools of similar scope and content [13,14,15,16,17,18,19,20]. We used the Reach, Effectiveness, Adoption, Implementation, and Maintenance (RE-AIM) Framework, a framework used to evaluate the implementation of evidence-informed interventions, and the Template for Intervention Description and Replication (TIDieR) checklist, a checklist that describes components that should be included when discussing new interventions, to inform the development of survey questions [21,22].

**Figure 1:**
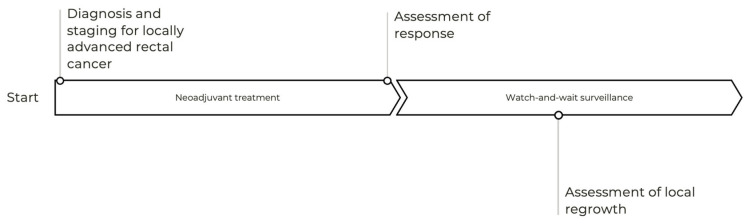
Terminology used to describe organ preservation for locally advanced rectal cancer.

To standardize the responses, we defined the key terms (Figure 1). The definitions were consistent with the International Watch and Wait Database and the International Consensus Recommendations on Key Outcome Measures for Organ Preservation After (Chemo) Radiation in Patients with Rectal Cancer [5]. “Primary organ preservation” was defined as a neoadjuvant therapy explicitly aimed at achieving a cCR or nCR to avoid surgery. “Secondary organ preservation” was defined as a neoadjuvant therapy administered with the conventional plan for TME, but when a cCR or nCR unexpectedly arises, surgery is deferred. Additional definitions can be found in Appendix A Table A1.

We piloted the survey among stakeholders with sufficient expertise who were not part of the final sample, refining content, flow, and clarity from April to May 2023 [23]. The finalized survey contained about 40 possible questions and required approximately 20 min to complete for those who saw the maximum number of items (Supplementary File S1).

### 2.4. Survey Administration

We distributed the final survey via the LimeSurvey platform from June to July 2023. An initial email invitation was sent, followed by three bi-weekly reminders. The invitations were in English and French for participants in Quebec, who indicated their preferred language. All Quebec participants selected English, so a French version was not ultimately required. The survey remained open for ten weeks.

### 2.5. Data Analysis

Given the study’s descriptive focus and the sample size, we employed descriptive statistics. Categorical variables are expressed as frequencies and percentages, while continuous variables appear as means with standard deviations (SD) or medians with interquartile ranges (IQR), depending on their distribution. Missing data are presented as a separate category where relevant.

## 3. Results

### 3.1. Respondent Characteristics

We received responses from 40 of 44 centers (91% response rate; Table 1). The respondents included radiation oncologists (24, 60.0%), surgeons (13, 32.5%), and medical oncologists (3, 7.5%) from all ten provinces. The respondents were from university-affiliated tertiary/quaternary centers (21, 52.5%), university-affiliated community centers (16, 40.0%), and non-university-affiliated community centers (3, 7.5%). The respondents were in practice for a median of eight years (IQR: 5–15) and saw a median of four (IQR: 3–6) new rectal cancer consults per month. 

### 3.2. Implementation of Organ Preservation 

**Figure 2:**
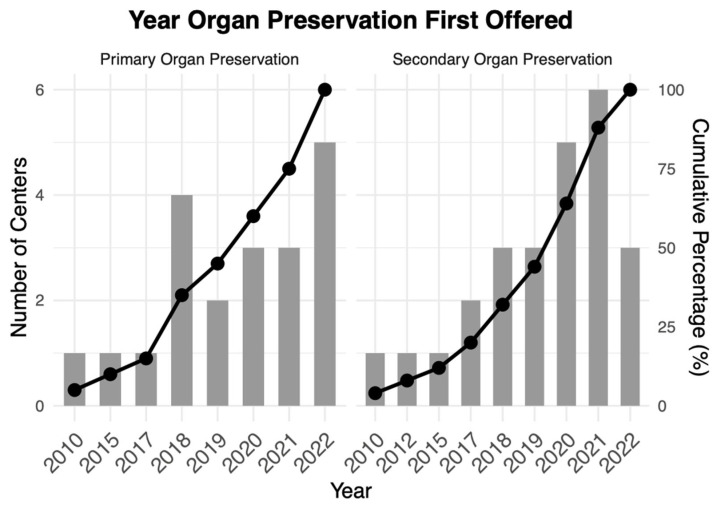
Adoption of primary* and secondary** organ preservation over time. This figure presents the introduction of primary and secondary organ preservation across centers over time. The gray bars represent the number of centers that first offered organ preservation each year. The black line represents the cumulative percentage of centers adopting organ preservation by that year. * = All 20 centers offering primary organ preservation responded. ** = Only 25/31 centers offering secondary organ preservation responded. Therefore, there are six missing responses from the secondary organ preservation group.

Organ preservation was offered in 31 (77.5%) centers (Table 1). Twenty (50.0%) respondents reported that their center offered primary and secondary organ preservation, 11 (27.8%) offered only secondary organ preservation, and eight (20.0%) offered neither. (Table 1). Primary and secondary organ preservation were first reported to be offered in 2010 (Figure 2). The median reported year that centers began offering primary organ preservation was 2020 (IQR 2018–2022), and for secondary organ preservation, 2020 (IQR 2018–2021; Table 1).

Among the twenty respondents from centers offering primary organ preservation, all twenty (100.0%) reported being involved in patient care for primary organ preservation, nine (45.0%) participated in developing primary organ preservation programs, five (25.0%) were institutional leaders and six (30.0%) conducted research in primary organ preservation.

Among twenty centers offering primary organ preservation, seven (35.0%) integrated it into standard practice, six (30.0%) offered it only upon patient request, eight (40.0%) only when a patient refused surgery, and five (25.0%) as part of the research. 

Among the nineteen respondents who reported not offering primary organ preservation, eight (42.1%) noted plans for future implementation, four (21.1%) had no plans for implementation, and seven (36.8%) reported their center had implemented alternative strategies (e.g., centralized organ preservation to a regional referral center, only offered to highly selected patients).

### 3.3. Variability in Patient Selection for Organ Preservation

Among the twenty centers offering primary organ preservation, patients were selected for primary organ preservation by their surgeon (15, 75.0%), multidisciplinary case conference (12, 60.0%), immediate treating team (6, 30.0%), medical oncologist (1, 5.0%) and/or radiation oncologist (1, 5.0%).

Regarding multidisciplinary case conferences (MCC), 12 (60.0%) centers indicated that 81–100% of patients considered for primary organ preservation were discussed at an MCC before starting neoadjuvant treatment (Table 2). After completing neoadjuvant treatment and assessing the treatment response, 10 (50.0%) centers reviewed 81–100% of these patients at a subsequent MCC (Table 2).

**Figure 3:**
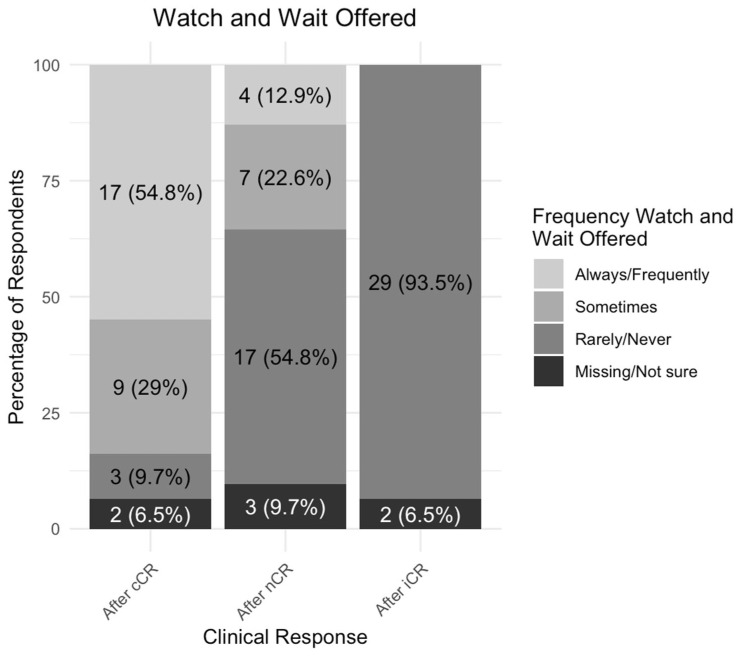
Proportion of patients offered a watch-and-wait surveillance following clinical response (*n* = 31) *. cCR = clinical complete response, nCR = near-complete response, iCR = incomplete clinical response. * = Among centers where primary and/or secondary organ preservation is offered (*n* = 31).

Among the 31 centers offering primary and/or secondary organ preservation, patients were more frequently offered watch-and-wait surveillance when they achieved a cCR (17, 54.8%) than a nCR (4, 12.9%; Figure 3).

### 3.4. Variability in Neoadjuvant Treatment Strategies in Organ Preservation 

Among the 20 centers offering primary organ preservation, the most common treatment strategies used when planning for primary organ preservation included chemoradiation followed by consolidation chemotherapy (i.e., OPRA—consolidation; 17, 89.5%) and long-course chemoradiation (6, 31.6%; Supplementary Table S1). Induction chemotherapy followed by chemoradiation (i.e., OPRA—induction; 2, 10.5%), triplet induction chemotherapy followed by chemoradiation (i.e., PRODIGE 23; 1, 5.3%), short-course radiotherapy followed by consolidation chemotherapy (i.e., RAPIDO; 2, 10.5%), and immunotherapy (0) were less commonly used (Supplementary Table S1).

The same patterns were seen among 31 centers where secondary organ preservation led to watch and wait. Most respondents cited long-course chemoradiation or chemoradiation plus consolidation chemotherapy as the regimen that yielded a complete clinical response (Supplementary Table S1).

### 3.5. Variability in the Assessment of Tumor Response and Watch-and-Wait Surveillance 

**Figure 4:**
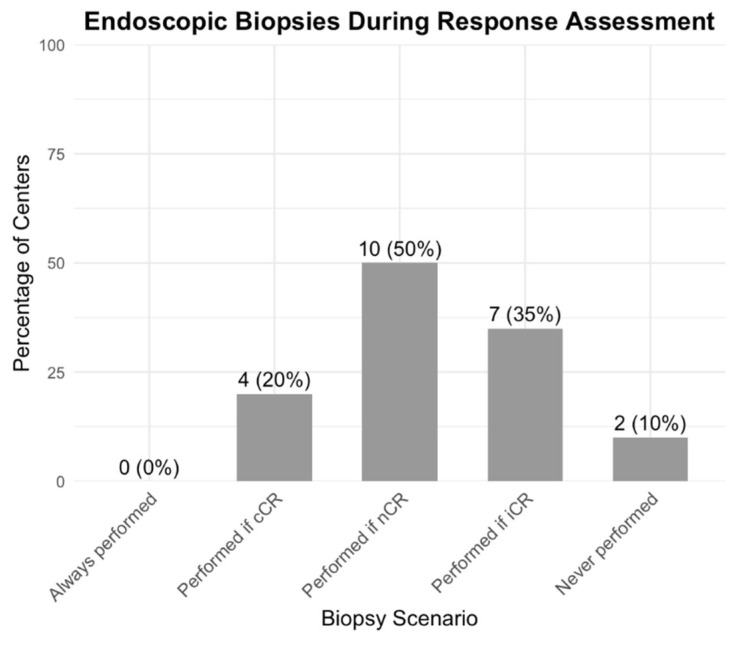
Frequency of endoscopic biopsies during response assessment for primary organ preservation (*n* = 20) *. cCR =clinical complete response; nCR = near-complete response; iCR = incomplete response. * = Among centers offering primary organ preservation (*n* = 20). Two responses were excluded from the figure as the respondents were unsure when the biopsies were performed, and one response was excluded as their response was missing.

Among the twenty centers offering primary organ preservation, tumor response was more often assessed after completing neoadjuvant therapy (19, 95.0%) than between treatments (7, 35.0%). Common modalities included pelvic MRI (19, 95.0%), flexible endoscopy (17, 85.0%), CT abdomen/pelvis (16, 80.0%), CT chest (15, 75.0%), and DRE (16, 80.0%; Supplementary Table S2). Rigid proctosigmoidoscopy (7, 35.0%) and CEA (12, 60.0%) were less frequently used. Tumor biopsies were performed selectively based on the clinical response (Figure 4). Modalities for watch-and-wait surveillance mirrored those used for an initial response assessment (Supplementary Table S2).

Among the twenty centers offering primary organ perseveration, six (30%) participants reported using clinical trial protocols to guide assessment of response, nine (45%) relied on international consensus guidelines, and eight (40.0%) relied on locoregional expert opinion. Among the 31 centers offering primary and/or secondary organ preservation, 13 (41.9%) participants reported using clinical trial protocols to guide watch-and-wait surveillance, 15 (48.4%) relied on international consensus guidelines, and 14 (35.0%) relied on locoregional expert opinion (Supplementary Table S3).

Among the 20 centers offering primary organ preservation, the operating surgeon most commonly performed endoscopy for response assessments in 17 centers (85.0%; Supplementary Table S2). The operating surgeon was also primarily responsible for organizing imaging investigations for response assessment (13, 68%; Supplementary Table S2). This was similar during the watch-and-wait surveillance among centers offering primary and/or secondary organ preservation (Supplementary Table S2). 

Among the 20 centers offering primary organ preservation, standardized criteria for determining a clinical response were more commonly available for MRI (18, 90.0%) than for endoscopy (12, 60.0%; Supplementary Table S2). Synoptic reporting templates for a clinical response were more frequently used for MRI (12, 60.0%) than endoscopy (1, 5.0%; Supplementary Table S2). This was similar during the watch-and-wait surveillance among the centers offering primary and/or secondary organ preservation (Supplementary Table S2).

### 3.6. Variability in Resources for Quality Assurance for Organ Preservation

Among the 31 centers offering primary and/or secondary organ preservation, quality assurance resources for healthcare professionals included group (26, 83.9%) and individual learning opportunities (18, 58.1%) and performance assessments (9, 29.0%). There were fewer resources available for patients. Psychosocial support services were available in 11 (35.5%) centers, clinical navigators were available in 8 (25.8%) centers, home care services were available in 5 centers, and patient education resources were available in 5 (16.1%) centers. The quality of care for patients pursuing primary and/or secondary organ preservation was monitored and evaluated prospectively in six (19.4%) centers and retrospectively in three (9.7%) centers. Eleven (35.5%) centers had plans to implement a system to monitor and evaluate the quality, and eleven (35.5%) centers had not made plans or were unsure of the plans to implement quality assurance for organ preservation. 

### 3.7. Challenges and Areas for Improvement 

Among all 40 respondents, the most commonly reported challenges to implementing primary organ preservation were access to MRI (21, 52.5%), clinic time (18, 45.0%), timely surgery if required (16, 40.0%), and lack of comfort/familiarity with the strength of supporting evidence (15, 37.5%) (Table 3). Open-ended comments for improvement were provided by 31 of the 40 respondents across five areas: (1) protocols, resources, and guidelines (14, 45.2%); (2) research and evidence (12, 38.7%); (3) collaboration (7, 22.6%); (4) patient engagement (6, 19.4%); and (5) healthcare professional knowledge (4, 12.9%). Illustrative quotes from the open-ended comments are included.

“Need more standardized surveillance guidelines, and likely more resources (i.e., MRI and endoscopy time) to dedicate to this approach.”

“A consolidated clinical pathway with defined roles and a clinical navigator.”

“Create formalized programs through policy/procedures for patients undergoing primary or secondary organ preservation.”

“Templates for reporting and formal guidelines adoptable from both larger and peripheral centers.”

“Communication between hospitals and patient travel makes this a challenge.”

## 4. Discussion 

This national survey provides the first comprehensive overview of organ preservation adoption for locally advanced rectal cancer in Canada. Our findings reveal that while organ preservation is recognized and increasingly offered, actual implementation is inconsistent across centers. Only around half of the respondents indicated their center offered primary organ preservation, and even fewer had fully integrated it into routine care pathways. Several factors may account for this limited and variable uptake.

Primary and secondary organ preservation began in Canada in 2010, nearly a decade after the approach was first described [24]. Steadily increasing evidence likely spurred expanded interest, with many centers implementing organ preservation in the past few years [25,26,27]. However, the gap between initial reports and clinical integration illustrates the complexity of adopting new treatment paradigms, particularly those requiring close coordination among multiple specialties and more resource-intensive follow-up.

Surgeons were primarily responsible for determining candidacy for primary organ preservation, with a less frequent, routine MCC review for all cases. Optimal selection requires input from a multidisciplinary team to account for tumor factors, patient preferences, and comorbidities. National quality initiatives in rectal cancer demonstrate the importance of multidisciplinary collaboration in improving outcomes [28,29,30]. A systematic MCC discussion of all potential organ-preservation candidates might yield more standardized evaluations and consensus-driven treatment plans, particularly when the local expertise or comfort level varies.

Respondents generally reported using a combination of MRI, endoscopy, and digital rectal examination to determine cCR or nCR, which aligns with best practices [5,31]. However, the practical challenge arises in consistently and accurately interpreting these tests. Biopsies were sometimes performed, even though they can yield false-negative results and are not universally recommended [32,33,34]. Inconsistent approaches to biopsy likely reflect the broader absence of standardized surveillance protocols. 

An accurate assessment of the treatment response and regrowth requires frequent, coordinated clinical, endoscopic, and radiological evaluations that are interpreted by experienced clinicians. Inter-rater variability remains a major barrier to the wider adoption of organ preservation [35]. Several standardized schemas (e.g., MSKCC, OPRA) aim to improve reproducibility, but they have yet to undergo extensive validation. Potential discrepancies in clinical response can significantly impact a patient and determine whether or not they will be offered organ preservation. Quality assurance is essential, yet few centers in our survey reported prospectively evaluating assessment quality. Synoptic endoscopy and MRI reports using standardized criteria can improve consistency and data quality but remain underused in Canada.

Since our survey was conducted in 2023, two prospective trials were published that further strengthened the evidence for organ preservation in rectal cancer. The phase II OPRA trial demonstrated that TNT, particularly with chemoradiation followed by consolidation chemotherapy, resulted in long-term organ preservation in half of the patients [36]. Similarly, the phase III OPERA trial demonstrated that brachytherapy after chemoradiotherapy resulted in organ preservation rates of up to 80% [37]. In addition, the 12-month outcomes of STAR-TREC were recently released in abstract form at the European Society of Radiotherapy and Oncology Annual Congress 2025, demonstrating that SCRT achieved 12-month TME-free survival rates of 61.5%, whereas CRT resulted in 12-month TME-free survival rates of 79.8% [38]. Finally, the pre-planned interim safety analysis and preliminary results of MORPHEUS were published in 2022, demonstrating that brachytherapy following chemoradiotherapy resulted in a 76.6% two-year TME-free survival [39]. The final results of this trial are pending publication. These studies further support that carefully selected patients can safely undergo organ preservation.

Recent international trials and society guidelines support the feasibility of implementing watch-and-wait strategies with structured follow-up protocols. The ESMO guidelines recommend MRI, endoscopy, and DRE every 3 months for the first 2 years and every 6 months thereafter, with chest and abdominal CT every 6 months for 2 years, then annually [6]. The NCCN guidelines similarly advise close monitoring with physical exam and CEA every 3–6 months, DRE and sigmoidoscopy every 3–4 months, rectal MRI every 6 months for up to 3 years, and colonoscopy 1 year following the completion of therapy [9]. While ASCRS emphasizes the need for more prospective data, it supports watch-and-wait in highly selected patients within protocolized settings, particularly with intensive surveillance in the first 2–3 years, when the risk of local regrowth is highest [7]. The 2024 ASCO guidelines standardize assessment by recommending evaluation for cCR at 8 ± 4 weeks after completion of TNT using specific criteria across DRE, rectoscopy, and MRI [8]. Notably, an endoscopic biopsy is not required and is discouraged if the clinical and radiologic features of cCR are fulfilled. The surveillance protocol for nonoperative management in this guideline follows the OPRA trial: DRE and flexible sigmoidoscopy every 4 months for the first 2 years from the time of assessment of the response, continuing every 6 months for the following 3 years, in addition to MRI every 6 months for the first 2 years, and yearly for the following 3 years. Collectively, these guidelines provide increasingly consistent definitions and follow-up strategies to support the safe implementation of nonoperative management.

The updated watch-and-wait surveillance protocols outlined in the recently published guidelines are informed by findings from contemporary clinical trials and protocols for locally advanced rectal cancer. The follow-up strategies described in MORPHEUS, OPRA, OPERA, and STAR-TREC vary slightly in intensity, timing, and imaging modalities for patients managed with organ preservation. MORPHEUS and OPERA adopt a more intensive schedule, with clinical and endoscopic evaluations every 3 months for the first two to three years, while OPRA follows a slightly less frequent schedule with assessments every four months initially [36,37,39]. MRI is obtained every 3 to 6 months in the first two years in all trials, though OPERA uses the most frequent imaging (every 3 months), and STAR-TREC applies fixed time points rather than regular intervals. CT imaging is included annually for five years in MORPHEUS and OPRA, but is less frequently in STAR-TREC, and is not clearly specified in OPERA [36,37,39,40]. These differences reflect varying thresholds for monitoring intensity in watch-and-wait strategies.

Although Canada does not currently have national guidelines for the implementation of watch-and-wait strategies in rectal cancer, we collected data on what sources clinicians rely on when making decisions regarding clinical response assessment and surveillance protocols. Our findings reflect the current heterogeneity of practice across Canadian centers and underscore the need for the development of a national consensus or adaptation of international frameworks to guide the standardization of organ preservation in Canada.

Respondents identified resource constraints as a major challenge, including insufficient MRI availability, limited endoscopy slots, and inadequate clinical time for frequent follow-ups. Although organ preservation is considered cost-effective due to avoided surgical morbidity [41,42,43], up-front resource investment remains crucial. Additional staff, more frequent imaging, and dedicated appointment times can strain capacity, particularly at smaller community centers.

The survey comments also suggested that formal institutional programs with dedicated coordinators or navigators might help streamline follow-ups, schedule imaging more systematically, and enhance patient support. Collaboration among centers may be necessary, particularly for regions with low volumes, to pool expertise and resources for organ preservation.

Although this study is Canadian, the obstacles are broadly relevant. In the United States, for instance, rectal cancer care is often delivered across multiple private clinics or hospitals, making consistent multidisciplinary collaboration and timely follow-ups challenging [44]. Access to imaging and surgical expertise can vary significantly by geographic area and insurance coverage [45]. In European countries with universal healthcare systems, existing centralization may ease some barriers, but issues like inter-rater variability, patient adherence, and limited dedicated funding persist [46]. Across diverse health systems, ensuring sufficient expertise, infrastructure, and patient support is vital for safe and effective organ preservation.

Respondents selected multiple areas for improvement and provided open-ended responses that suggested multiple ways to address implementation barriers. Many participants emphasized standardized clinical protocols and surveillance pathways, including dedicated institutional programs and clinical navigators, to streamline care. Enhanced access to imaging and endoscopy through regional scheduling or protected slots was deemed essential. Improved collaboration among centers, especially for smaller or remote sites, could reduce disparities. Overall, these insights indicate that strengthening system-level infrastructure, inter-professional communication, and shared decision-making frameworks is key to achieving consistent and equitable organ preservation.

## 5. Limitations

Our use of a key-informant approach may not reflect every nuance of individual practice at each center. We surveyed only one informant per center, raising the possibility of specialty-specific biases. However, we deliberately selected key informants because they were well-positioned to provide a comprehensive overview of organ preservation practices at their respective institutions, offering insights that extend beyond their own clinical activities. This approach allowed us to gather high-level information on institutional norms and capacity. Moreover, the use of key informants is a well-established and widely accepted sampling strategy in qualitative and exploratory research [47].

The participants were not evenly distributed among medical, radiation, and surgical oncologists. Given that organ preservation strategies most directly influence decisions around radiation therapy and surgical intervention, we anticipated that most key informants would be from these specialties. We contacted an average of two to three physicians per center before identifying a key informant, indicating that our snowball sampling strategy extended beyond immediate professional networks and allowed for broader engagement within each institution. 

Because the data were self-reported, recall bias is possible. We attempted to minimize misinterpretations by providing clear definitions and piloting the survey. Our approach captures practices at a single point in time (June–July 2023) and may not reflect continual shifts or upcoming expansions. However, it is unlikely that practice has changed significantly since this survey was conducted. Furthermore, patient perspectives on accessibility and experience with organ preservation were not addressed, as this was outside the scope of our research question. However, patient insights could offer valuable context regarding the perceived accessibility of organ preservation strategies at the local level and represent an important area for future investigation.

Our results may not be fully generalizable beyond Canada’s publicly funded, regionalized healthcare system. Nonetheless, the core lessons on resource needs, clinical coordination, and standardized protocols can inform similar efforts internationally.

## 6. Conclusions

Organ preservation for locally advanced rectal cancer has gained acceptance in Canada since 2010, but implementation remains inconsistent, with only half of the centers offering it. Multidisciplinary input is often lacking, limiting optimal decision-making. Although evidence supports the safety and cost-effectiveness of organ preservation, its successful implementation hinges on adequate resources for intensive monitoring and prompt salvage, which are not universally available in Canada. Wider use of standardized response criteria, synoptic reporting, and prospective quality evaluations could improve consistency in assessing clinical responses and help expand equitable access to organ preservation in Canada’s publicly funded healthcare system.

## Figures and Tables

**Table 1:** Characteristics of survey respondents (*n* = 40).

Characteristics	*n* (%) or Median (IQR)
Geographical Region	
Atlantic (NFL, PEI, NS, NB)	4 (10.0%)
Quebec	10 (25.0%)
Ontario	15 (37.5%)
Prairies (MB, SK, AB)	5 (12.5%)
British Columbia	6 (15.0%)
Type of Radiation Center	
Non-university-affiliated community center	3 (7.5%)
University-affiliated community center	16 (40.0%)
University-affiliated tertiary/quaternary center	21 (52.5%)
Specialty	
Medical oncology	3 (7.5%)
Radiation oncology	24 (60.0%)
Surgery	13 (32.5%)
Clinical Experience	
Years in practice	8 (5–15)
Year started practice at current institution	2015 (2008–2018)
Number of rectal cancer consults per month	4 (3–6)
Resources for Managing Locally Advanced Rectal Cancer	
MRI	40 (100.0%)
CT	40 (100.0%)
Endoscopy	39 (97.5%)
EUS	8 (20.0%)
PET Scan	6 (15.0%)
GI-specialized pathology	30 (75.0%)
GI-specialized medical oncology	39 (97.5%)
GI-specialized radiation oncology	39 (97.5%)
Rectal cancer clinical trials	19 (47.5%)
AYA program	1 (2.5%)
Psychosocial oncology program	17 (42.5%)
Laparoscopy	32 (80.0%)
Robotic	2 (5.0%)
MCC Frequency	
Every two weeks	14 (35.0%)
Weekly	26 (65.0%)
Patients with Locally Advanced Rectal Cancer Discussed at MCC Before Neoadjuvant Treatment	
0–20%	2 (5.0%)
21–40%	5 (12.5%)
41–60%	2 (5.0%)
61–80%	8 (20.0%)
81–100%	23 (57.5%)
Patients with Locally Advanced Rectal Cancer Discussed at MCC After Neoadjuvant Treatment and Before Surgery	
0–20%	20 (50.0%)
21–40%	2 (5.0%)
41–60%	6 (15.0%)
61–80%	6 (15.0%)
81–100%	4 (10.0%)
Missing	2 (5.0%)
Status of Organ Preservation	
Primary and secondary organ preservation are offered	20 (50.0%)
Secondary organ preservation is offered only	11 (27.8%)
Organ preservation is not offered	8 (20.0%)
Missing	1 (2.5%)
Year Organ Preservation First Offered	
Primary and secondary organ preservation	2020 (2018–2022)
Secondary organ preservation	2020 (2018–2021)

AYA = adolescents and young adults; AB = Alberta; CT = computed tomography; EUS = endoscopic ultrasound; GI = gastrointestinal; IQR = interquartile range; MB = Manitoba; MCC = multidisciplinary case conference; MRI = magnetic resonance imaging; NB = New Brunswick; NFL = Newfoundland and Labrador; NS = Nova Scotia; PEI = Prince Edward Island; PET = positron emission tomography; SK = Saskatchewan.

**Table 2:** Patients undergoing primary organ preservation discussed at multidisciplinary case conference (*n* = 20) *.

Category	*n* (%)
Before Neoadjuvant Treatment	
0–20%	1 (5.0%)
21–40%	2 (10.0%)
41–60%	2 (10.0%)
61–80%	3 (15.0%)
81–100%	12 (60.0%)
After Assessment of Response	
0–20%	5 (25.0%)
21–40%	1 (5.0%)
41–60%	3 (15.0%)
61–80%	1 (5.0%)
81–100%	10 (50.0%)

* = Among centers where primary organ preservation is offered (*n* = 20).

**Table 3:** Challenges and areas for improvement perceived with primary organ preservation for locally advanced rectal cancer (*n* = 40).

Perceived Challenges (Select All That Apply)	*n* (%)
Access to MRI	21 (52.5%)
Clinic time/space for the number of required assessments	18 (45.0%)
Access to timely surgery if required	16 (40.0%)
Lack of comfort/familiarity with long-term outcomes of supporting evidence	15 (37.5%)
Staff capacity for the number of required assessments	14 (35.0%)
Access to endoscopy	13 (32.5%)
Lack of comfort/familiarity with the strength of supporting evidence	13 (32.5%)
Access to the cancer center	12 (30.0%)
Lack of comfort/familiarity with the clinical and radiological assessment and surveillance protocols	11 (27.5%)
Suboptimal coordination among multidisciplinary team	10 (25.0%)
Lack of comfort/familiarity with the necessary treatment protocols	7 (17.5%)
Missing	2 (5.0%)

## Data Availability

The original contributions presented in this study are included in the article/Supplementary Material. Further inquiries can be directed to the corresponding author(s).

## Supplementary Materials

The following supporting information can be downloaded at: https://www.mdpi.com/article/10.3390/curroncol32060341/s1, Table S1: Treatment strategy used when planning for primary organ preservation and among those eligible for watch-and-wait strategies; Table S2: Investigations used for response assessment and watch-and-wait surveillance in patients undergoing primary and secondary organ preservation; Table S3: Factors that guide the determination of a patient’s assessment of response, and subsequent watch-and-wait surveillance protocol.

## Appendix A

**Table A1:** Definitions of Key Terms Used in the Survey to Describe Organ Preservation.

Key Term	Definition
Assessment of response	The use of clinical, radiological and/or pathological investigations after completion of therapy in primary organ preservation to determine their eligibility for watch-and-wait surveillance vs. surgical management
Clinical complete response	No residual disease, as determined through clinical, endoscopic, and radiological assessments
Clinical incomplete response	Minimal or no response to treatment, with clear evidence of residual tumor in the bowel wall or mesorectal nodes.
Clinical near-complete response	Substantial treatment response that does not fully meet the criteria for a clinical complete response.
Locally advanced rectal cancer	Stage II/III, T1-2N+ or T3/4N any
Primary organ preservation	A treatment strategy where neoadjuvant therapy is administered with the explicit goal of achieving a clinical complete or near-complete response, thereby avoiding total mesorectal excision
Secondary organ preservation	A treatment strategy where neoadjuvant therapy is administered and a complete clinical response or near-complete clinical response is achieved, thereby avoiding total mesorectal excision, despite the primary aim of neoadjuvant treatment being to proceed with surgery
Watch and wait surveillance	A strict surveillance protocol following a clinical complete or near-complete response, applicable to both primary and secondary organ preservation
