# Supplementary material for: Implementation of Organ Preservation for Locally Advanced Rectal Cancer in Canada: A National Survey of Clinical Practice
Source: Curr Oncol. 2025 Jun 10;32(6):341. doi: 10.3390/curroncol32060341 (PMC12191898; doi:10.3390/curroncol32060341)
Supplement: Supplementary file 1 [file curroncol-32-00341-s001.zip › curroncol-3656942-supplementary.pdf]

Supplementary Table 1: Treatment strategy used when planning for primary organ preservation and among those eligible for watch and wait

	Always/Frequently n (%)	Sometimes n (%)	Rarely/Never n (%)	Missing/Not sure n (%)
**Treatment strategy used when planning for primary organ preservation (n=20)**				
OPRA - consolidation	17 (85.0%)	2 (10%)	0 (0.0%)	1 (5.0%)
OPRA - induction	2 (10%)	9 (45.0%)	8 (40.0%)	1 (5.0%)
PRODIGE 23	1 (5.0%)	3 (15.0%)	15 (75.0%)	1 (5.0%)
RAPIDO	2 (10.0%)	7 (35.0%)	9 (45.0%)	1 (5.0%)
Short-course radiotherapy	0 (0.0%)	2 (10.0%)	15 (75.0%)	1 (5.0%)
Long-course chemoradiotherapy	6 (30.0%)	7 (35.0%)	7 (35.0%)	1 (5.0%)
Immunotherapy	0 (0.0%)	1 (5.0%)	17 (85.0%)	2 (10.0%)
**Treatment strategy used among those eligible for watch and wait (n=31)**				
OPRA - consolidation	17 (54.8%)	6 (19.4%)	4 (12.9%)	4 (12.9%)
OPRA - induction	5 (16.1%)	5 (16.1%)	16 (51.6%)	5 (16.1%)
PRODIGE 23	1 (3.2%)	5 (16.1%)	20 (64.5%)	5 (16.1%)
RAPIDO	5 (16.1%)	14 (45.2%)	8 (25.8%)	4 (12.9%)
Short-course radiotherapy	0 (0.0%)	4 (12.9%)	21 (67.7%)	6 (19.4%)
Long-course chemoradiotherapy	5 (16.1%)	6 (19.4%)	15 (48.4%)	5 (16.1%)
Immunotherapy	0 (0.0%)	1 (3.2%)	24 (77.4%)	6 (19.4%)

Footnote: OPRA - Consolidation refers to standard long-course chemoradiotherapy followed by consolidation chemotherapy. OPRA - Induction refers to induction chemotherapy followed by long-course chemoradiotherapy. PRODIGE 23 refers to triplet induction chemotherapy (FOLFIRINOX) followed by long-course chemoradiotherapy. RAPIDO refers to short-course radiotherapy followed by consolidation chemotherapy.

Supplementary Table 2: Investigations Used for Response Assessment and Watch and Wait Surveillance in Patients Undergoing Primary and Secondary Organ Preservation

**Investigations used for assessment of response after neoadjuvant treatment in patients undergoing primary organ preservation (select all that apply, n=20)**	
Pelvic MRI	19 (95.0%)
Flexible endoscopy	17 (85.0%)
Rigid proctosigmoidoscopy	7 (35.0%)
CT chest	15 (75.0%)
CT abdomen/pelvis	16 (80.0%)
PET CT	0 (0.0%)
DRE	16 (80.0%)
CEA	12 (60.0%)
Missing	1 (5.0%)
**Investigations used For watch and wait surveillance in patients undergoing primary and secondary organ preservation (select all that apply, n=31)**	
Pelvic MRI	29 (93.5%)
Flexible endoscopy	27 (87.1%)
Rigid proctosigmoidoscopy	11 (35.5%)
CT chest	22 (71.0%)
CT abdomen/pelvis	25 (80.6%)

PET CT	1 (3.2%)
DRE	25 (80.6%)
CEA	24 (77.4%)
Missing	1 (6.5%)
**Endoscopist most commonly performing assessment of response (n=20)**	
Operating surgeon	17 (85.0%)
Any colorectal surgeon	1 (5.0%)
Any surgeon	0 (0.0%)
Gastroenterology	1 (5.0%)
Missing	1 (5.0%)
**Most responsible for organizing imaging for assessment of response among those offering primary organ preservation (n=20)**	
Shared responsibility	7 (35.0%)
Nurse or allied health professional	1 (5.0%)
Medical oncologist	4 (20.0%)
Radiation oncologist	5 (25.0%)
Operating surgeon	13 (65.0%)
Missing	1 (5.0%)
**Endoscopist most commonly performing watch and watch surveillance (n=31)**	

Operating surgeon	25 (80.6%)
Any colorectal surgeon	4 (12.9%)
Any surgeon	1 (3.2%)
Gastroenterology	2 (6.5%)
Missing	3 (9.7%)
**Most responsible for organizing imaging for watch and wait among those offering primary and secondary organ preservation (n=31)**	
Shared responsibility	7 (22.6%)
Nurse or allied health professional	3 (9.7%)
Medical oncologist	3 (9.7%)
Radiation oncologist	4 (12.9%)
Operating surgeon	22 (71.0%)
Missing	2 (6.5%)
**Standardized criteria and dedicated templates (i.e., synoptic report) used for assessment of response (select all that apply, n= 20)**	
Standardized MRI criteria	18 (90.0%)
Dedicated MRI template (i.e., synoptic report)	12 (60.0%)
Standardized endoscopy criteria	12 (60.0%)
Dedicated endoscopy template (i.e., synoptic report)	1 (5.0%)
**Standardized criteria and dedicated templates (i.e., synoptic report) used for assessing local regrowth on watch and wait (select all that apply, n= 31)**	

Standardized MRI criteria	22 (71.0%)
Dedicated MRI template (i.e., synoptic report)	12 (38.7%)
Standardized endoscopy criteria	15 (48.4%)
Dedicated endoscopy template (i.e., synoptic report)	1 (3.2%)
**Frequency of photo-documentation during the assessment of response on endoscopy when cCR suspected (total n=20)**	
Always/frequently	11 (55.0%)
Sometimes	1 (5.0%)
Never	1 (5.0%)
Not sure	4 (2.0%)
Missing	3 (15.0%)
**Frequency of photo-documentation during the assessment of response on endoscopy when nCR suspected (total n=20)**	
Always/frequently	11 (55.0%)
Sometimes	1 (5.0%)
Never	1 (5.0%)
Not sure	4 (2.0%)
Missing	3 (15.0%)
**Frequency of photo-documentation during the assessment of response on endoscopy when iCR suspected (total n=20)**	
Always/frequently	9 (45.0%)

Sometimes	2 (10.0%)
Never	1 (5.0%)
Not sure	5 (25.0%)
Missing	3 (15.0%)
**Frequency of photo-documentation during watch and wait on endoscopy when local regrowth not suspected(n=31)**	
Always/frequently	13 (41.9%)
Sometimes	4 (12.9%)
Never	1 (3.2%)
Not sure	8 (25.8%)
Missing	6 (19.4%)
**Frequency of photo-documentation during watch and wait on endoscopy when local regrowth suspected (n=31)**	
Always/frequently	14 (45.2%)
Sometimes	2 (6.5%)
Never	1 (3.2%)
Not sure	10 (32.3%)
Missing	5 (16.1%)

Footnote: cCR =clinical complete response; iCR = incomplete response; nCR = near complete response; MRI = Magnetic Resonance Imaging; CT = Computed Tomography; PET CT = Positron Emission Tomography Computed Tomography; DRE = Digital Rectal Examination; CEA = Carcinoembryonic Antigen.

Supplementary table 3: Factors that guide the determination of a patient’s assessment of response, and subsequent watch and wait surveillance protocol

**Factor**		**Assessment of response n (%) (n = 20)**	**Watch and wait surveillance protocol n (%) (n = 31)**
Clinical trial protocols	Yes<br>Missing	6 (30.0%)<br>1 (5.0%)	13 (41.9%)<br>1 (5.0%)
International consensus guidelines	Yes<br>Missing	9 (45.0%)<br>1 (5.0%)	15 (48.4%)<br>1 (5.0%)
Locoregional expert opinion	Yes<br>Missing	8 (40.0%)<br>1 (5.0%)	14 (35.0%)<br>1 (5.0%)

Supplementary file 1: Survey

Organ Preservation for Locally Advanced Rectal Cancer

IMPLIED CONSENT FORM

STUDY TITLE

Organ Preservation for Locally Advanced Rectal Cancer in Canada: A Survey of Real-World Clinical Practice

PRINCIPAL INVESTIGATOR

Kristopher Dennis MD PhD FRCPC
Radiation Oncologist
Radiation Medicine Program, The Ottawa Hospital
Associate Professor
Division of Radiation Oncology, University of Ottawa
501 Smyth Rd, Ottawa, Ontario, Canada, K1H8L6
Tel: 613-737-7700x70212
Fax: 613-247-3511
krdennis@toh.ca

OHSN-REB #
20230275-01H

INTRODUCTION

You are being asked to participate because you are a clinician involved in the management of patients with locally advanced rectal cancer. This study is an online survey examining organ preservation for locally advanced rectal cancer in real-world clinical practice in Canada.

This information sheet provides you with details to help you make an informed choice about participation in this survey. Please read this document carefully and ask any questions you may have. All your questions should be answered to your satisfaction before you decide whether to participate in this research study.

Please take your time in making your decision. Taking part in this study is voluntary. Your decision will not affect your employment. You have the option to not participate at all or you may choose to leave the study at any time.

ARE THERE ANY CONFLICTS OF INTEREST?

There are no conflicts of interest to declare related to this study.

WHAT WILL HAPPEN DURING THIS STUDY?
Your participation in this study will require the completion of an online survey. The survey will take a maximum of 20 minutes to complete. The questions on the survey ask about your practice and institutional characteristics and your experience with organ preservation for locally advanced rectal cancer.

The information you provide is for research purposes only. Some of the questions are personal. You can choose not to answer questions if you wish.

Communication via e-mail is not absolutely secure. We do not recommend that you communicate sensitive personal information via e-mail.

VOLUNTARY PARTICIPATION AND WITHDRAWAL
You do not have to be in this study if you do not want to be. You can choose to end your participation in this research (called withdrawal) at any time without having to provide a reason. The decision will not affect your employment. You can withdraw from participating at any time while completing the survey/questionnaire simply by closing your browser or not returning the hardcopy document; however, once the completed survey/questionnaire has been returned to the study team, it will not be possible to withdraw your information. Any information recorded before you withdraw will be used by the researchers for the purposes of the study, but no information will be collected after you withdraw your permission.

RISKS AND/OR BENEFITS

Participation involves minimal risk to you. Some of the questions may however make you feel uncomfortable. You may feel more fatigued after answering this survey.

You may not receive direct benefit from participating in this study. We hope the information learned from this study will help other people who use organ preservation for locally advanced rectal cancer in the future.

PRIVACY/CONFIDENTIATLITY

The survey will be anonymized which means that your answers will not be linked to you in any way. Authorized representatives of the following organizations may look at your original research records at the site where these records are held, to check that the information collected for the study is correct and follows proper laws and guidelines:

- The Ottawa Health Science Network Research Ethics Board who oversees the ethical conduct of this study.
- Ottawa Hospital Research Institute to oversee the conduct of research at this location.

Information that is collected about you for the study (called study data) may also be sent to the organizations listed above.

If the results of this study are published, your identity will remain confidential. It is expected that the information collected during this study will be used in analyses and will be published/ presented to the scientific community at meetings and in journals.

Your anonymized data from this study may be used for other research purposes. If your study data is shared with other researchers, information that links your study data directly to you will not be shared. Even though the risk of identifying you from the study data is very small, it can never be completely eliminated.

COST AND/OR PAYMENT

You will not be paid for being in this study, nor will there be any cost to you.

RIGHTS OF PARTICIPANTS

You will be told, in a timely manner, about new information that may be relevant to your willingness to stay in this study. You have the right to be informed of the results of this study once the entire study is complete. If you would like to be informed of the results of this study, please contact the research team.

Your rights to privacy are legally protected by federal and provincial laws that require safeguards to ensure that your privacy is respected.

QUESTIONS

If you have any questions about taking part in this study, you may contact the Principal Investigator, Dr. Kristopher Dennis, at 613-737-7700 ext. 70212.

If you have questions about your rights as a participant or about ethical issues related to this study, you can talk to someone who is not involved in the study at all. Please contact The Ottawa Health Science Network Research Ethics Board, Chairperson at 613-798-5555 extension 16719.

CONSENT

By completing this survey your consent to participate is implied.
There are 50 questions in this survey.

Clinical Practice Information

Answer the following questions based **on the typical practice patterns** at the institution(s) where you are currently working **predominantly with cancer patients**.

In what province do you currently work?

Choose one of the following answers
Please choose **only one** of the following:

- ◯ Alberta
- ◯ British Columbia
- ◯ Manitoba
- ◯ New Brunswick
- ◯ Newfoundland and Labardor
- ◯ Nova Scotia
- ◯ Ontario
- ◯ Prince Edward Island
- ◯ Quebec
- ◯ Saskatchewan

Which best describes the institution where you currently work?

❗ Choose one of the following answers

Please choose **only one** of the following:

- ◯ Community hospital
- ◯ University-affiliated community hospital
- ◯ University-affiliated tertiary/quaternary hospital
- ◯ Other

What is the name of the institution where you currently work?

Please write your answer here:

What is your main clinical specialty?

❗ Choose one of the following answers

Please choose **only one** of the following:

- ◯ Surgery
- ◯ Radiation oncology
- ◯ Medical oncology
- ◯ Other

What year did you start working **independently** in your main clinical specialty **at your curent institution**?

Please write your answer here:

How many years have you been working **independently** in your main clinical specialty at **any institution**?

Please write your answer here:

What is the average number of new consultations for rectal cancer you personally see per month?

Please write your answer here:

[ ]

Clinical Practice Information

Answer the following questions based **on the typical practice patterns** at the institution(s) where you are currently working **predominantly with cancer patients**.

Consider the following definitions:
Locally advanced rectal cancer: Stage II/III, T1-2N+ or T3/4Nany.

Which of the following resources are **typically utilized** for managing patients with **locally advanced rectal cancer**?

Check all that apply
Please choose **all** that apply:

- [ ] MRI
- [ ] PET CT
- [ ] Endorectal US
- [ ] CT
- [ ] Endoscopy
- [ ] Advanced endoscopy (e.g., endoscopic mucosal resection +/- endoscopic submucosal dissection)
- [ ] Laparoscopic colorectal surgery
- [ ] Robotic colorectal surgery
- [ ] Transanal Endoscopic Microsurgery (TEM) and/or Transanal Minimally Invasive Surgery (TAMIS)
- [ ] Medical oncologist specialized in rectal cancer
- [ ] Radiation oncologist specialized in rectal cancer
- [ ] Gastroenterology
- [ ] Interventional radiology
- [ ] Clinical trials for rectal cancer
- [ ] Genetics
- [ ] Pathology review
- [ ] Adolescent and Young Adult (AYA) cancer program
- [ ] Psychosocial oncology program (i.e., emotional, social, and psychological support and care)
- [ ] Other: [ ]

Locally advanced rectal cancer*: Stage II/III, T1-2N+ or T3/4Nany.*

How frequently do multidisciplinary case conferences occur where rectal cancer cases are discussed?

❶ Choose one of the following answers
Please choose **only one** of the following:

- ◯ Never
- ◯ Weekly
- ◯ Every 2 weeks
- ◯ Monthly
- ◯ Other [ ]

***Locally advanced rectal cancer**: Stage II/III, T1-2N+ or T3/4Nany*

What is the typical format used for multidisciplinary case conferences to discuss rectal cancer cases?

Only answer this question if the following conditions are met:
Answer was 'Weekly' *or* 'Monthly' *or* 'Every 2 weeks' at question ' [G02Q09]' (How frequently do multidisciplinary case conferences occur where rectal cancer cases are discussed?)

❶ Check all that apply
Please choose **all** that apply:

- ☐ Videoconference (e.g., Microsoft Teams, Zoom, etc.)
- ☐ In person
- ☐ Other: [ ]

What percentage of patients with **locally advanced rectal cancer** do you estimate are discussed in multidisciplinary case conferences in the following scenarios:

Only answer this question if the following conditions are met:
((G02Q09.NAOK (/questionAdministration/view/surveyid/336177/gid/11/qid/711) == "AO02" or G02Q09.NAOK (/questionAdministration/view/surveyid/336177/gid/11/qid/711) == "AO03" or G02Q09.NAOK (/questionAdministration/view/surveyid/336177/gid/11/qid/711) == "AO04"))

Please choose the appropriate response for each item:

	Not sure	0-20%	21-40%	41-60%	61-80%	81-100%
**Before any neoadjuvant treatment**	◯	◯	◯	◯	◯	◯
**After any neoadjuvant treatment but before surgery and/or organ preservation**	◯	◯	◯	◯	◯	◯

***Locally advanced rectal cancer**: Stage II/III, T1-2N+ or T3/4Nany*

Organ Preservation for Locally Advanced Rectal Cancer

Answer the following questions based **on the typical practice patterns** at the institution(s) where you are currently working **predominantly with cancer patients**.

Consider the following definitions:
Locally advanced rectal cancer: Stage II/III, T1-2N+ or T3/4Nany.

Primary organ preservation: The treatment strategy where neoadjuvant treatment is given **with the explicit goal of achieving a clinical complete/near-complete response** and avoiding total mesorectal excision.

Secondary organ preservation: The treatment strategy where neoadjuvant treatment is given **without the initial explicit goal of achieving a clinical complete/near-complete response** and avoiding total mesorectal excision, but patients transition to a watch and wait surveillance protocol after completing neoadjuvant treatment based on a satisfactory clinical response on assessment.

Clinical complete response: No residual disease when assessed by clinical, endoscopic and radiological studies.

Clinical near complete response: Do not fulfill the criteria of a clinical complete response but present a substantial response to treatment.

Clinical incomplete response: Minimal or no response to treatment with unequivocal residual tumor in the bowel wall or the mesorectal nodes.

Is **primary and/or secondary organ preservation** currently offered as a treatment for **locally advanced rectal cancer**? *

❶ Choose one of the following answers
Please choose **only one** of the following:

- ◯ Yes, primary organ preservation
- ◯ Yes, secondary organ preservation
- ◯ Yes, primary and secondary organ preservation
- ◯ No

To the best of your knowledge, in which year was **primary organ preservation** for **locally advanced rectal cancer** first offered?

Only answer this question if the following conditions are met:
((G03Q12.NAOK (/questionAdministration/view/surveyid/336177/gid/12/qid/714) == "AO01") or (G03Q12.NAOK (/questionAdministration/view/surveyid/336177/gid/12/qid/714) == "AO03"))

Please write your answer here:

__Primary organ preservation__: The treatment strategy where neoadjuvant treatment is given __with the explicit goal of achieving a complete/near-complete clinical response__ and avoiding total mesorectal excision.

What is the current status of **primary organ preservation** as a treatment option for **locally advanced rectal cancer** patients?

Only answer this question if the following conditions are met:
((G03Q12.NAOK (/questionAdministration/view/surveyid/336177/gid/12/qid/714) == "AO04") or (G03Q12.NAOK (/questionAdministration/view/surveyid/336177/gid/12/qid/714) == "AO02"))

❗ Choose one of the following answers
Please choose **only one** of the following:

- ◯ At stage of implementation
- ◯ Planning to implement in the future
- ◯ Not planning to implement in the future
- ◯ I am not familiar with primary organ preservation
- ◯ Other [ ]

Locally advanced rectal cancer: Stage II/III, T1-2N+ or T3/4Nany.

Primary organ preservation: *The treatment strategy where neoadjuvant treatment is given* ***with the explicit goal of achieving a complete/near-complete clinical response*** *and avoiding total mesorectal excision.*

To the best of your knowledge, in what year was **secondary organ preservation** for **locally advanced rectal cancer** first offered?

Only answer this question if the following conditions are met:
((G03Q12.NAOK (/questionAdministration/view/surveyid/336177/gid/12/qid/714) == "AO02") or (G03Q12.NAOK (/questionAdministration/view/surveyid/336177/gid/12/qid/714) == "AO03"))

Please write your answer here:

[ ]

Secondary organ preservation: *The treatment strategy where neoadjuvant treatment is given* ***without the initial explicit goal of achieving a complete/near-complete clinical response*** *and avoiding total mesorectal excision, but patients transition to a watch and wait surveillance protocol after completing neoadjuvant treatment based on a satisfactory clinical response on assessment.*

Which activities are commonly used by healthcare professionals to support the provision of primary and/or secondary organ preservation?

Only answer this question if the following conditions are met:
((G03Q12.NAOK (/questionAdministration/view/surveyid/336177/gid/12/qid/714) == "AO01") or (G03Q12.NAOK (/questionAdministration/view/surveyid/336177/gid/12/qid/714) == "AO02") or (G03Q12.NAOK (/questionAdministration/view/surveyid/336177/gid/12/qid/714) == "AO03"))

❗ Check all that apply
Please choose **all** that apply:

- ☐ Group learning activities (e.g., conferences, seminars, presentations, workshops)
- ☐ Individual learning activities (e.g., formal courses, research, quality improvement)
- ☐ Performance assessments (e.g., feedback, audits, direct observation)
- ☐ Other: [ ]

Healthcare professionals include doctors, nurses, physician assistants, pharmacists, therapists, and other allied health professionals.

Which resources are commonly used to support patients who undergo primary and/or secondary organ preservation?

Only answer this question if the following conditions are met:
((G03Q12.NAOK (/questionAdministration/view/surveyid/336177/gid/12/qid/714) == "AO01") or (G03Q12.NAOK (/questionAdministration/view/surveyid/336177/gid/12/qid/714) == "AO02") or (G03Q12.NAOK (/questionAdministration/view/surveyid/336177/gid/12/qid/714) == "AO03"))

❗ Check all that apply
Please choose **all** that apply:

- ☐ Educational material specific to primary organ preservation treatment
- ☐ Psychosocial support services (e.g., social work, physiotherapy, dietician, psychology, etc.)
- ☐ Clinical navigator
- ☐ Home care services (e.g., nursing, personal support workers, etc.)
- ☐ Other: ____________

Primary Organ Preservation for Locally Advanced Rectal Cancer

Answer the following questions based **on the typical practice patterns** at the institution(s) where you are currently working **predominantly with cancer patients**.

Consider the following definitions:

Locally advanced rectal cancer: Stage II/III, T1-2N+ or T3/4Nany.

Primary organ preservation: The treatment strategy where neoadjuvant treatment is given **with the explicit goal of achieving a clinical complete/near-complete response** and avoiding total mesorectal excision.

Secondary organ preservation: The treatment strategy where neoadjuvant treatment is given **without the initial explicit goal of achieving a clinical complete/near-complete response** and avoiding total mesorectal excision, but patients transition to a watch and wait surveillance protocol after completing neoadjuvant treatment based on a satisfactory clinical response on assessment.

Clinical complete response: No residual disease when assessed by clinical, endoscopic and radiological studies.

Clinical near complete response: Do not fulfill the criteria of a clinical complete response but present a substantial response to treatment.

Clinical incomplete response: Minimal or no response to treatment with unequivocal residual tumor in the bowel wall or the mesorectal nodes.

In which of the following scenarios is **primary organ preservation** offered to patients with **locally advanced rectal cancer**:

❶ Check all that apply

Please choose **all** that apply:

- [ ] It is part of routine clinical care
- [ ] It is part of a defined protocol or pathway
- [ ] Some clinicians offer it individually but not all
- [ ] It is only offered if a patient requests it
- [ ] It is only offered if a patient refuses the recommended surgery
- [ ] It is only offered as part of a research study (e.g., prospective registry, clinical trial, etc.)
- [ ] Other: ____________

Consider only the scenario where a patient with **locally advanced rectal cancer** would be **medically suitable for both surgery and organ preservation.**

What is your role(s) in providing **primary organ preservation** for **locally advanced rectal cancer**?

❶ Check all that apply

Please choose **all** that apply:

- [ ] I provide care to patients who are receiving primary organ preservation.
- [ ] I participate in the development of a primary organ preservation program.
- [ ] I am a leader of a primary organ preservation program, overseeing patient care and program development.
- [ ] I am involved in primary organ preservation as part of research.
- [ ] Other: ____________

Primary organ preservation: *The treatment strategy where neoadjuvant treatment is given* ***with the explicit goal of achieving a complete/near-complete clinical response*** *and avoiding total mesorectal excision.*

What percentage of patients with **locally advanced rectal cancer undergoing primary organ preservation** do you estimate are discussed in multidisciplinary case conferences in the following scenarios:

Please choose the appropriate response for each item:

	Not sure	0-20%	21-40%	41-60%	61-80%	81-100%
**Before neoadjuvant treatment for primary organ preservation**	○	○	○	○	○	○
**After assessment of response to neoadjuvant treatment for primary organ preservation**	○	○	○	○	○	○

Primary organ preservation: *The treatment strategy where neoadjuvant treatment is given **with the explicit goal of achieving a complete/near-complete clinical response** and avoiding total mesorectal excision.*

Assessment of response: *Clinical, radiological and/or pathological investigations used to determine a patient's clinical response after **completion of all neoadjuvant therapy**. This is used in patient's **planning for primary organ preservation** to determine their eligibility for a watch and wait surveillance protocol versus total mesorectal excision.*

How commonly are the following neoadjuvant treatments used in patients **who are planning** for **primary organ preservation**?

Please choose the appropriate response for each item:

	Not sure	Never	Rarely	Sometimes	Frequently	Always
**Long-course chemoradiotherapy followed by consolidation FOLFOX (e.g., OPRA)**	○	○	○	○	○	○
**Induction FOLFOX followed by long-course chemoradiotherapy (e.g., OPRA)**	○	○	○	○	○	○
**Induction FOLFIRINOX followed by long-course chemoradiotherapy (e.g., PRODIGE 23)**	○	○	○	○	○	○
**Short-course radiotherapy followed by consolidation chemotherapy (e.g., RAPIDO)**	○	○	○	○	○	○
**Short-course radiotherapy**	○	○	○	○	○	○
**Long-course chemoradiotherapy**	○	○	○	○	○	○
**Immunotherapy alone**	○	○	○	○	○	○
**Cytotoxic therapy alone**	○	○	○	○	○	○
**Targeted therapy alone**	○	○	○	○	○	○

Primary organ preservation: *The treatment strategy where neoadjuvant treatment is given **with the explicit goal of achieving a complete/near-complete clinical response** and avoiding total mesorectal excision.*

Which **tumor factors** typically determine a patient' s eligibility for **primary organ preservation** (e.g., T/N stage, location, CRM, need for alternative surgery, etc.)?

Please write your answer here:

Consider only the scenario where a patient with **locally advanced rectal cancer** would be **medically suitable for both surgery and organ preservation.**

Assessment of Response After Neoadjuvant Treatment for Primary Organ Preservation

Answer the following questions based **on the typical practice patterns** at the institution(s) where you are currently working **predominantly with cancer patients**.

Consider the following definitions:

Locally advanced rectal cancer: Stage II/III, T1-2N+ or T3/4Nany.

Primary organ preservation: The treatment strategy where neoadjuvant treatment is given **with the explicit goal of achieving a clinical complete/near-complete response** and avoiding total mesorectal excision.

Secondary organ preservation: The treatment strategy where neoadjuvant treatment is given **without the initial explicit goal of achieving a clinical complete/near-complete response** and avoiding total mesorectal excision, but patients transition to a watch and wait surveillance protocol after completing neoadjuvant treatment based on a satisfactory clinical response on assessment.

Clinical complete response: No residual disease when assessed by clinical, endoscopic and radiological studies.

Clinical near complete response: Do not fulfill the criteria of a clinical complete response but present a substantial response to treatment.

Clinical incomplete response: Minimal or no response to treatment with unequivocal residual tumor in the bowel wall or the mesorectal nodes.

Assessment of response: Clinical, radiological and/or pathological investigations used to determine a patient's clinical response after **completion of all neoadjuvant therapy**. This is used in patient's **planning for primary organ preservation** to determine their eligibility for a watch and wait surveillance protocol versus total mesorectal excision.

How frequently is the **<u>assessment of response</u>** to neoadjuvant treatment typically performed in patients pursuing primary organ preservation in the following scenarios:

Please choose the appropriate response for each item:

	Not sure	Never	Rarely	Sometimes	Frequently	Always
**Between phases of neoadjuvant treatment (e.g., between long-course chemoradiotherapy and consolidation chemotherapy)**	○	○	○	○	○	○
**After completing all neoadjuvant treatment**	○	○	○	○	○	○

Assessment of response: Clinical, radiological and/or pathological investigations used to determine a patient's clinical response after **completion of all neoadjuvant therapy**. This is used in patient's **planning for primary organ preservation** to determine their eligibility for watch and wait versus total mesorectal excision.

Which of the following investigations are commonly used for the assessment of resonse?

❶ Check all that apply

Please choose **all** that apply:

- ☐ Pelvic MRI
- ☐ Flexible endoscopy
- ☐ Rigid proctosigmoidoscopy
- ☐ CT chest
- ☐ CT abdomen/pelvis
- ☐ PET-CT
- ☐ Digital rectal examination
- ☐ CEA
- ☐ Other: ____________

Assessment of response: *Clinical, radiological and/or pathological investigations used to determine a patient's clinical response after* ***completion of all neoadjuvant therapy****. This is used in patient's* ***planning for primary organ preservation*** *to determine their eligibility for a watch and wait surveillance protocol versus total mesorectal excision.*

In what scenarios is a biopsy commonly performed of the primary tumor during the assessment of response?

Check all that apply
Please choose **all** that apply:

- [ ] Always
- [ ] Clinical complete response suspected
- [ ] Clinical near complete response suspected
- [ ] Clinical incomplete response suspected
- [ ] Never
- [ ] Not sure
- [ ] Other: ______

Assessment of response: *Clinical, radiological and/or pathological investigations used to determine a patient's clinical response after* ***completion of all neoadjuvant therapy****. This is used in patient's* ***planning for primary organ preservation*** *to determine their eligibility for a watch and wait surveillance protocol versus total mesorectal excision.*

Clinical complete response: *No residual disease when assessed by clinical, endoscopic and radiological studies.*

Clinical near complete response: *Do not fulfill the criteria of a clinical complete response but present a substantial response to treatment.*

Clinical incomplete response: *Minimal or no response to treatment with unequivocal residual tumor in the bowel wall or the mesorectal nodes.*

Who is most commonly responsible for organizing **IMAGING** for the **assessment of response**?

Only answer this question if the following conditions are met:
((G05Q26_SQ001 (/questionAdministration/view/surveyid/336177/gid/15/qid/728) == "Y") or (G05Q26_SQ004 (/questionAdministration/view/surveyid/336177/gid/15/qid/728) == "Y") or (G05Q26_SQ005 (/questionAdministration/view/surveyid/336177/gid/15/qid/728) == "Y") or (G05Q26_SQ006 (/questionAdministration/view/surveyid/336177/gid/15/qid/728) == "Y"))

Check all that apply
Please choose **all** that apply:

- [ ] Shared responsibility aided by secure communications between providers
- [ ] Advanced practice nurse or equivalent dedicated allied health staff
- [ ] Medical oncology
- [ ] Radiation oncology
- [ ] Last specialist to actively treat patient
- [ ] The surgeon who would be performing the patient's surgery
- [ ] Any surgeon who can perform rectal cancer surgery (e.g., group practice model)
- [ ] Surgeons who do not routinely perform rectal cancer surgery (e.g., community general surgeon)
- [ ] Gastroenterologists
- [ ] It is not clear who is primarily responsible
- [ ] Other: ______

Assessment of response: *Clinical, radiological and/or pathological investigations used to determine a patient's clinical response after* ***completion of all neoadjuvant therapy****. This is used in patient's* ***planning for primary organ preservation*** *to determine their eligibility for a watch and wait surveillance protocol versus total mesorectal excision.*

Are **standard MRI criteria** (e.g., a signal on diffusion-weighted or T2 imaging) used for the **assessment of response**?

Only answer this question if the following conditions are met:
(G05Q26_SQ001 (/questionAdministration/view/surveyid/336177/gid/15/qid/728) == "Y")

❶ Choose one of the following answers
Please choose **only one** of the following:

- ◯ Yes
- ◯ No
- ◯ Not sure

Assessment of response: *Clinical, radiological and/or pathological investigations used to determine a patient's clinical response after* ***completion of all neoadjuvant therapy****. This is used in patient's* ***planning for primary organ preservation*** *to determine their eligibility for watch and wait versus total mesorectal excision.*

Is a **dedicated template** (i.e., synoptic report) used to document a patient's clinical response on **MRI**?

Only answer this question if the following conditions are met:
(G05Q26_SQ001 (/questionAdministration/view/surveyid/336177/gid/15/qid/728) == "Y")

❶ Choose one of the following answers
Please choose **only one** of the following:

- ◯ Yes
- ◯ No
- ◯ Not sure

Synoptic report: *A concise, standardized electronic or paper report included in a patient's medical record that includes all the necessary data for the assessment of response.*

Assessment of response: *Clinical, radiological and/or pathological investigations used to determine a patient's clinical response after* ***completion of all neoadjuvant therapy****. This is used in patient's* ***planning for primary organ preservation*** *to determine their eligibility for a watch and wait surveillance protocol versus total mesorectal excision.*

Who most commonly performs **endoscop y** for the **assessment of response**?

Only answer this question if the following conditions are met:
((G05Q26_SQ002 (/questionAdministration/view/surveyid/336177/gid/15/qid/728) == "Y") or (G05Q26_SQ003 (/questionAdministration/view/surveyid/336177/gid/15/qid/728) == "Y"))

❶ Check all that apply
Please choose **all** that apply:

- [ ] The surgeon who would be performing the patient's surgery
- [ ] Any surgeon who can perform rectal cancer surgery (e.g., group practice model)
- [ ] Surgeons who do not routinely perform rectal cancer surgery (e.g., community general surgeon)
- [ ] Gastroenterologists
- [ ] Other: ____________

Assessment of response: *Clinical, radiological and/or pathological investigations used to determine a patient's clinical response after* ***completion of all neoadjuvant therapy****. This is used in patient's* ***planning for primary organ preservation*** *to determine their eligibility for a watch and wait surveillance protocol versus total mesorectal excision.*

Are **standard endoscopic criteria** (e.g., scar, ulceration nodularity) used for the **assessment of response**?

Only answer this question if the following conditions are met:
((G05Q26_SQ002.NAOK (/questionAdministration/view/surveyid/336177/gid/15/qid/728) == "Y") or (G05Q26_SQ003.NAOK (/questionAdministration/view/surveyid/336177/gid/15/qid/728) == "Y"))

❶ Choose one of the following answers
Please choose **only one** of the following:

- ( ) Yes
- ( ) No
- ( ) Not sure

Assessment of response: *Clinical, radiological and/or pathological investigations used to determine a patient's clinical response after* ***completion of all neoadjuvant therapy****. This is used in patient's* ***planning for primary organ preservation*** *to determine their eligibility for a watch and wait surveillance protocol versus total mesorectal excision.*

Is a **dedicated template** (i.e., synoptic report) used to document a patient's clinical response on **endoscop y**?

Only answer this question if the following conditions are met:
((G05Q26_SQ002.NAOK (/questionAdministration/view/surveyid/336177/gid/15/qid/728) == "Y") or (G05Q26_SQ003.NAOK (/questionAdministration/view/surveyid/336177/gid/15/qid/728) == "Y"))

❗ Choose one of the following answers
Please choose **only one** of the following:

- ◯ Yes
- ◯ No
- ◯ Not sure

Synoptic report: *A concise, standardized electronic or paper report included in a patient's medical record that includes all the necessary data for the assessment of response.*

Assessment of response: *Clinical, radiological and/or pathological investigations used to determine a patient's clinical response after* ***completion of all neoadjuvant therapy****. This is used in patient's* ***planning for primary organ preservation*** *to determine their eligibility for a watch and wait surveillance protocol versus total mesorectal excision.*

How frequently is **photo documentation** used during **flexible endoscop y** in the following scenarios?

Only answer this question if the following conditions are met:
(G05Q26_SQ002.NAOK (/questionAdministration/view/surveyid/336177/gid/15/qid/728) == "Y")

Please choose the appropriate response for each item:

	Not sure	Never	Rarely	Sometimes	Frequently	Always
**Clinical complete response suspected**	◯	◯	◯	◯	◯	◯
**Clinical near complete response suspected**	◯	◯	◯	◯	◯	◯
**Clinical incomplete response suspected**	◯	◯	◯	◯	◯	◯

Assessment of response: *Clinical, radiological and/or pathological investigations used to determine a patient's clinical response after* ***completion of all neoadjuvant therapy****. This is used in patient's* ***planning for primary organ preservation*** *to determine their eligibility for watch and wait versus total mesorectal excision.*

Who most commonly determines a patient's clinical response and eligibility for a watch and wait surveillance protocol versus surgery?

❶ Check all that apply
Please choose **all** that apply:

- [ ] Surgeon
- [ ] Multidisciplinary case conference members
- [ ] Multidisciplinary treating team (i.e., not including all case conference members)
- [ ] Medical oncologist
- [ ] Radiation oncologist
- [ ] Other: ______

Assessment of response: *Clinical, radiological and/or pathological investigations used to determine a patient's clinical response after* ***completion of all neoadjuvant therapy****. This is used in patient's* ***planning for primary organ preservation*** *to determine their eligibility for a watch and wait surveillance protocol versus total mesorectal excision.*

How is a patient's clinical response and eligibility for a watch and wait surveillance protocol versus surgery communicated?

❶ Check all that apply
Please choose **all** that apply:

- [ ] Multidisciplinary case conference discussion
- [ ] Direct communication (e.g., secure messaging among multidisciplinary team)
- [ ] Indirect communication (e.g., multidisciplinary team notes in patient chart)
- [ ] Other: ______

Assessment of response: *Clinical, radiological and/or pathological investigations used to determine a patient's clinical response after* ***completion of all neoadjuvant therapy****. This is used in patient's* ***planning for primary organ preservation*** *to determine their eligibility for a watch and wait surveillance protocol versus total mesorectal excision.*

What guides the determination of a patient's clinical response and eligibility for a watch and wait surveillance protocol versus surgery?

❶ Check all that apply
Please choose **all** that apply:

- [ ] Clinical trials (e.g., OPRA)
- [ ] International consensus recommendations or guidelines
- [ ] Local/regional expert recommendations or guidelines
- [ ] None of these
- [ ] Other: ______

Assessment of response: *Clinical, radiological and/or pathological investigations used to determine a patient's clinical response after* ***completion of all neoadjuvant therapy****. This is used in patient's* ***planning for primary organ preservation*** *to determine their eligibility for a watch and wait surveillance protocol versus total mesorectal excision.*

Watch and Wait for Locally Advanced Rectal Cancer

Answer the following questions based **on the typical practice patterns** at the institution(s) where you are currently working **predominantly with cancer patients**.

Please consider the following definitions:

Primary organ preservation: The treatment strategy where neoadjuvant treatment is given **with the explicit goal of achieving a clinical complete/near-complete response** and avoiding total mesorectal excision.

Secondary organ preservation: The treatment strategy where neoadjuvant treatment is given **without the initial explicit goal of achieving a clinical complete/near-complete response** and avoiding total mesorectal excision, but patients transition to a watch and wait surveillance protocol after completing neoadjuvant treatment based on a satisfactory clinical response on assessment.

Watch and wait: A strict surveillance protocol after achieving a complete clinical response or near complete clinical response. This protocol can be used in primary or secondary organ preservation.

Local regrowth: Reappearance, clinically, radiologically and/or pathogically, of overt adenocarcinoma in the mucosa or deeper layers of the rectal wall, or in mesorectal/lateral nodes **after the achievement of a complete clinical response or near complete clinical response**.

How often is **watch and wait** an option for patients in each of the following scenarios?

Please choose the appropriate response for each item:

	Not sure	Never	Rarely	Sometimes	Frequently	Always
**Clinical complete response**	○	○	○	○	○	○
**Clinical near complete response**	○	○	○	○	○	○
**Clinical incomplete response**	○	○	○	○	○	○

Clinical complete response: *No residual disease when assessed by clinical, endoscopic and radiological studies.*

Clinical near complete response: *Do not fulfill the criteria of a clinical complete response but present a substantial response to treatment.*

Clinical incomplete response: *Minimal or no response to treatment with unequivocal residual tumor in the bowel wall or the mesorectal nodes.*

What guides the surveillance protocol used for watch and wait (e.g., timing and criteria/interpretation of investigations)?

❗ Check all that apply

Please choose **all** that apply:

- ☐ Clinical trials (e.g., OPRA)
- ☐ International consensus recommendations or guidelines
- ☐ Local/regional expert recommendations or guidelines
- ☐ None
- ☐ Other: __________

Watch and wait*: A strict surveillance protocol after achieving a complete clinical response or near complete clinical response. This protocol can be used in primary or secondary organ preservation.*

How commonly are the following neoadjuvant treatments used in patients **<u>who are eligible for a watch and wait surveillance protocol</u>**?

Please choose the appropriate response for each item:

	Not sure	Never	Rarely	Sometimes	Frequently	Always
**Long-course chemoradiation followed by consolidation FOLFOX (e.g., OPRA)**	◯	◯	◯	◯	◯	◯
**Induction FOLFOX followed by long-course chemoradiation (e.g., OPRA)**	◯	◯	◯	◯	◯	◯
**Induction FOLFIRINOX followed by long-course chemoradiation (e.g., PRODIGE 23)**	◯	◯	◯	◯	◯	◯
**Short-course radiation followed by consolidation chemotherapy (e.g., RAPIDO)**	◯	◯	◯	◯	◯	◯
**Short-course radiation**	◯	◯	◯	◯	◯	◯
**Long-course chemoradiation**	◯	◯	◯	◯	◯	◯
**Immunotherapy alone**	◯	◯	◯	◯	◯	◯
**Cytotoxic therapy alone**	◯	◯	◯	◯	◯	◯
**Targeted therapy alone**	◯	◯	◯	◯	◯	◯

<u>Watch and wait</u>*: A strict surveillance protocol after achieving a complete clinical response or near complete clinical response. This protcol can be used in primary or secondary organ preservation.*

Which of the following are commonly used in a watch and wait surveillance protocol?

❗ Check all that apply

Please choose **all** that apply:

- ☐ Pelvic MRI
- ☐ Flexible endoscopy
- ☐ Rigid proctosigmoidoscopy
- ☐ CT chest
- ☐ CT abdomen/pelvis
- ☐ PET-CT
- ☐ Digital rectal examination
- ☐ CEA
- ☐ Other: ________

<u>Watch and wait</u>*: A strict surveillance program after achieving a complete clinical response or near complete clinical response. This program can be used in primary or secondary organ preservation. The exact timing, methods/studies and criteria/interpretation used are entirely at the discretion of the participating centres.*

Who is most commonly responsible for organizing **IMAGING** for patients on a **watch and wait** surveillance protocol?

Only answer this question if the following conditions are met:
((G06Q41_SQ001 (/questionAdministration/view/surveyid/336177/gid/16/qid/743) == "Y") or (G06Q41_SQ004 (/questionAdministration/view/surveyid/336177/gid/16/qid/743) == "Y") or (G06Q41_SQ005 (/questionAdministration/view/surveyid/336177/gid/16/qid/743) == "Y") or (G06Q41_SQ006 (/questionAdministration/view/surveyid/336177/gid/16/qid/743) == "Y"))

❗ Check all that apply
Please choose **all** that apply:

- ☐ Shared responsibility aided by secure communications between providers
- ☐ Advanced practice nurse or equivalent dedicated allied health staff
- ☐ Medical oncology
- ☐ Radiation oncology
- ☐ The surgeon who would perform the patient's surgery
- ☐ Any surgeon who can perform rectal cancer surgery (e.g., group practice model)
- ☐ Surgeons who do not routinely perform rectal cancer surgery (e.g., community general surgery)
- ☐ Gastroenterologists
- ☐ Last specialist to actively treat patient
- ☐ Primary care provider
- ☐ Transitional cancer care clinic
- ☐ Not sure
- ☐ Other: ____________

Are **standard criteria** (e.g., a signal on diffusion-weighted or T2 imaging) used for the assessment of **local regrowth** on **MRI**?

Only answer this question if the following conditions are met:
(G06Q41_SQ001 (/questionAdministration/view/surveyid/336177/gid/16/qid/743) == "Y")

❗ Choose one of the following answers
Please choose **only one** of the following:

- ◯ Yes
- ◯ No
- ◯ Not sure

Watch and wait: *A strict surveillance program after achieving a complete clinical response or near complete clinical response. This program can be used in primary or secondary organ preservation.*

Local regrowth: *Reappearance, clinically or radiologically, of overt adenocarcinoma, biopsy confirmed if possible, in the mucosa or deeper layers of the rectal wall, or in mesorectal/lateral nodes after the achievement of a complete clinical response or near complete clinical response.*

Is there a **dedicated template** (e.g., synoptic report) to document the assessment of **local regrowth** for patients on a **watch and wait surveillance protocol**?

Only answer this question if the following conditions are met:
(G06Q41_SQ001 (/questionAdministration/view/surveyid/336177/gid/16/qid/743) == "Y")

❶ Choose one of the following answers
Please choose **only one** of the following:

- ◯ Yes
- ◯ No
- ◯ Not sure

***Synoptic report**: A concise, standardized electronic or paper report included in a patient's medical record that includes all the necessary data for the assessment of response.*

***Watch and wait**: A strict surveillance program after achieving a complete clinical response or near complete clinical response. This program can be used in primary or secondary organ preservation.*

***Local regrowth**: Reappearance, clinically or radiologically, of overt adenocarcinoma, biopsy confirmed if possible, in the mucosa or deeper layers of the rectal wall, or in mesorectal/lateral nodes after the achievement of a complete clinical response or near complete clinical response.*

Who is most commonly responsible for organizing **ENDOSCOPY** for patients on a watch and wait surveillance protocol?

Only answer this question if the following conditions are met:
((G06Q41_SQ002 (/questionAdministration/view/surveyid/336177/gid/16/qid/743) == "Y") or (G06Q41_SQ003 (/questionAdministration/view/surveyid/336177/gid/16/qid/743) == "Y"))

❶ Check all that apply
Please choose **all** that apply:

- ☐ Shared responsibility aided by secure communications between providers
- ☐ Advanced practice nurse or equivalent dedicated allied health staff
- ☐ Medical oncology
- ☐ Radiation oncology
- ☐ The surgeon who would perform the patient's surgery
- ☐ Any surgeon who can perform rectal cancer surgery (e.g., group practice model)
- ☐ Surgeons who do not routinely perform rectal cancer surgery (e.g., community general surgery)
- ☐ Gastroenterologists
- ☐ Last specialist to actively treat patient
- ☐ Primary care provider
- ☐ Transitional cancer care clinic
- ☐ Not sure
- ☐ Other: ____________

Are **standard criteria** (e.g., scar, ulceration nodularity) used for the assessment of **local regrowth** on endoscopy?

Only answer this question if the following conditions are met:
((G06Q41_SQ002 (/questionAdministration/view/surveyid/336177/gid/16/qid/743) == "Y") or (G06Q41_SQ003 (/questionAdministration/view/surveyid/336177/gid/16/qid/743) == "Y"))

❗ Choose one of the following answers
Please choose **only one** of the following:

- ◯ Yes
- ◯ No
- ◯ Not sure

Watch and wait*: A strict surveillance program after achieving a complete clinical response or near complete clinical response. This program can be used in primary or secondary organ preservation.*

Is a **dedicated template** (i.e., synoptic report) used to document the assessment of local regrowth on **endoscop y**?

Only answer this question if the following conditions are met:
((G06Q41_SQ002 (/questionAdministration/view/surveyid/336177/gid/16/qid/743) == "Y") or (G06Q41_SQ003 (/questionAdministration/view/surveyid/336177/gid/16/qid/743) == "Y"))

❗ Check all that apply
Please choose **all** that apply:

- ☐ Yes
- ☐ No
- ☐ Not sure

Synoptic report*: A concise, standardized electronic or paper report included in a patient's medical record that includes all the necessary data for the assessment of response.*

Watch and wait*: A strict surveillance program after achieving a complete clinical response or near complete clinical response. This program can be used in primary or secondary organ preservation.*

Local regrowth*: Reappearance, clinically or radiologically, of overt adenocarcinoma, biopsy confirmed if possible, in the mucosa or deeper layers of the rectal wall, or in mesorectal/lateral nodes after the achievement of a complete clinical response or near complete clinical response.*

How frequently is **photo documentation** used during **flexible endoscopy** in the following scenarios?

Only answer this question if the following conditions are met:
(G06Q41_SQ002 (/questionAdministration/view/surveyid/336177/gid/16/qid/743) == "Y")

Please choose the appropriate response for each item:

	Not sure	Never	Rarely	Sometimes	Frequently	Always
**Local regrowth suspected**	○	○	○	○	○	○
**Local regrowth not suspected**	○	○	○	○	○	○

Watch and wait: *A strict surveillance program after achieving a complete clinical response or near complete clinical response. This program can be used in primary or secondary organ preservation.*

Who most commonly performs endoscopy for patients on a **watch and wait surveillance protocol**?

Only answer this question if the following conditions are met:
((G06Q41_SQ002 (/questionAdministration/view/surveyid/336177/gid/16/qid/743) == "Y") or (G06Q41_SQ003 (/questionAdministration/view/surveyid/336177/gid/16/qid/743) == "Y"))

❗ Check all that apply
Please choose **all** that apply:

- ☐ The surgeon who would perform the patient's surgery
- ☐ Any surgeon who can perform rectal cancer surgery (e.g., group practice model)
- ☐ Surgeons who do not routinely perform rectal cancer surgery (e.g., community general surgery)
- ☐ Gastroenterologists
- ☐ Not sure
- ☐ Other: ______________________

Quality Assurance & Quality Improvement

Answer the following questions based **on the typical practice patterns** at the institution(s) where you are currently working **predominantly with cancer patients**.

Is the quality of care for patients pursuing primary or secondary organ preservation for locally advanced rectal cancer being monitored and evaluated?

Check all that apply

Please choose **all** that apply:

- ☐ Yes, prospectively
- ☐ Yes, retrospectively
- ☐ Not currently but planning to implement in the future
- ☐ Not currently and no plans to implement in the future
- ☐ Not sure
- ☐ Other: ____________

This can be formal or informal activities, including research or quality improvement.

Future Directions

Answer the following questions based **on the typical practice patterns** at the institution(s) where you are currently working **predominantly with cancer patients**.

Consider the following definitions:

Locally advanced rectal cancer: Stage II/III, T1-2N+ or T3/4Nany.

Primary organ preservation: The treatment strategy where neoadjuvant treatment is given **with the explicit goal of achieving a complete/near-complete clinical response** and avoiding total mesorectal excision.

What challenges do you perceive with **primary organ preservation** for **locally advanced rectal cancer**?

Comment only when you choose an answer.
Please choose all that apply and provide a comment:

- [ ] Access to endoscopy
- [ ] Access to MRI
- [ ] Access to cancer center (e.g. due to geography)
- [ ] Access to timely surgery if required
- [ ] Clinic time/space for the number of assessments required
- [ ] Staff capacity for the number of assessments required
- [ ] Suboptimal coordination among multidisciplinary team
- [ ] Lack of comfort/familiarity with strength of supporting evidence
- [ ] Lack of comfort/familiarity with longterm outcomes of supporting evidence
- [ ] Lack of comfort/familiarity with the necessary treatment protocols
- [ ] Lack of comfort/familiarity with the clinical and radiological assessment and surveillance protocols

Other:

You can choose to include any comments beside any option you select.

Describe any areas for improvement you perceive with **primary organ preservation** for **locally advanced rectal cancer**.

Please write your answer here:

Please consider the following definitions:

Locally advanced rectal cancer*: Stage II/III, T1-2N+ or T3/4Nany.*

Primary organ preservation: The treatment strategy where neoadjuvant treatment is given **with the explicit goal of achieving a complete/near-complete clinical response** and avoiding total mesorectal excision.

Submit your survey.
Thank you for completing this survey.
